# Is It Time to Alter the Standard of Care for Iron Deficiency/Iron Deficiency Anemia in Reproductive-Age Women?

**DOI:** 10.3390/biomedicines12020278

**Published:** 2024-01-25

**Authors:** Mrutyunjaya B. Bellad, Anmol Patted, Richard J. Derman

**Affiliations:** 1 Department of Obstetrics and Gynecology, KAHER’s Jawaharlal Nehru Medical College, Belagavi 590010, Karnataka, India; belladmb@gmail.com; 2 Department of Global Affairs, Thomas Jefferson University, Philadelphia, PA 19107, USA; anmol.patted@jefferson.edu

**Keywords:** iron deficiency, anemia, pregnancy, women, intravenous iron supplementation

## Abstract

Two billion people worldwide suffer from anemia, with reproductive-age women being disproportionately affected. Iron plays a crucial role in cellular function and impacts cognition, physical function, and quality of life. Iron deficiency (ID) and iron deficiency anemia (IDA) are associated with adverse effects on pregnancy and fetal development. Oral iron supplementation has been the standard treatment for decades, often producing sub-optimal outcomes. Many babies are still being born with ID and suffer adverse sequelae due to inadequate iron levels in the mothers. Is it time to consider a broad scale-up of parenteral iron as a new standard of care?

## 1. Introduction

Iron deficiency (ID) and anemia remain global public health challenges. Iron deficiency is the most common nutritional deficiency in the world [1,2], and one in four people globally suffer from anemia, a condition in which there is a lower than normal number of red blood cells (RBCs) or hemoglobin concentration (<130 g/L in males and <120 g/L in non-pregnant females) [3]. Inadequate iron stores impair the production of RBCs, resulting in iron deficiency anemia (IDA) [2], and the intricate interconnections among ID, IDA, and anemia often make it difficult to identify the root cause of its negative consequences. Reproductive-age women are disproportionately affected by ID/IDA [3], and this burden may be due to increased physiological demands during pregnancy and menstruation as well as socioeconomic factors resulting in poor nutrition and a lack of access to quality healthcare [4,5,6].

There is a wide variation in the prevalence of anemia in reproductive-age women across world regions. It ranges from 20% in the United States and Western Europe to 40–60% in sub-Saharan Africa and parts of Asia [3]. In 2011, the highest prevalence of anemia was reported in the low- and middle-income countries of Central and West Africa and South Asia [7]. The 2019–2021 National Family Health Survey-5 data from India reported that 67.1% of all children aged 6–59 months, 57.0% of reproductive-age women, and 52.2% of pregnant women were anemic, demonstrating that anemia is widely prevalent across different at-risk age groups [8]. Although the global prevalence of ID without anemia is not well established, the United States National Health and Nutrition Examination Survey (NHANES) reported a prevalence of 38.6% in non-pregnant women between 12 and 21 years of age [9]. Furthermore, ID is thought to be the underlying cause of anemia in approximately 45% of reproductive-age women in high-income countries and up to 80% of women in low- and middle-income countries [10].

## 2. Discussion

### 2.1. The Role of Iron in Cell Life and Its Homeostasis

The majority of the body’s iron is present in the hemoglobin of red blood cells. Iron is crucial for the mediation of various cellular functions, including respiration and oxygen transport employing iron-containing proteins such as cytochromes and hemoglobin, respectively. DNA synthesis and repair, mitochondrial function, and energy production: iron serves as co-factors for ribonucleotide reductase and several enzymes in oxidative phosphorylation and cell proliferation by interacting with various regulatory proteins and signaling pathways [11]. It also plays a critical role in synthesizing heme, myoglobin, cytochromes, and several enzymes, making it indispensable for life. It is imperative that the body maintains iron homeostasis, ensuring the smooth functioning of cells and leading to overall health.

The regulation of iron availability is a complex process, and hepcidin plays a central role in maintaining the systemic iron balance. Hepcidin is a protein that is primarily synthesized in the liver in response to tissue and circulating iron levels. It functions by binding to and inactivating ferroportin, the only known cellular iron export protein [12]. Hepcidin controls the absorption of iron from the gastrointestinal tract, its distribution throughout the body, and its storage in the form of ferritin when in excess [12]. High circulating serum iron levels, inflammation, and infection result in increased synthesis of hepcidin, while ID, tissue hypoxia, and increased erythropoiesis lead to decreased synthesis [12]. Given the closely regulated nature of iron levels, iron deficiency is often due to low intake of dietary iron and/or excessive iron loss associated with heavy menstrual bleeding or a present or previous pregnancy [13]. 

### 2.2. The Impact of Iron Deficiency, Iron Deficiency Anemia, and Anemia in Women of Reproductive Age

ID is associated with negative and adverse outcomes, including fatigue, decreased cognition, diminished physical function, sub-optimal development, and poor quality of life (QOL) [14,15,16]. A study by Bruner et al. evaluated the cognitive function of 81 adolescent female participants with documented ID, defined as a serum ferritin level ≤ 12 µg/L, in a double-blinded, placebo-controlled study on the effects of iron supplementation with an oral ferrous sulfate compound (650 mg twice daily) or placebo. The study assessed the cognitive function of the participants using “questionnaires and hematological and cognitive tests” at different intervals within an eight-week period and found that the participants in both the study arms had a similar learning curve at the baseline. However, the placebo-treated participants with ID scored lower than those who received iron supplementation at each successive administration of the Hopkins Verbal Learning Test [14].

Another double-blinded study by Rowland et al. examined the physical implications of ID in 14 iron-deficient female cross-country runners, stratified by initial serum ferritin level, who were randomly assigned to either receive iron supplementation with a ferrous sulfate compound (975 mg/day) or placebo after a 4-week control period. Their endurance time was measured by treadmill testing at the beginning of the season (week 0), at the end of the control period (week 4), and after treatment (week 8). The study team noted that the endurance time significantly declined in all placebo-treated subjects, while performance improved in those who received iron (*p* < 0.01) [15].

Patterson et al. investigated the association between self-reported ‘low iron’ and general health and fatigue by conducting a questionnaire-based study in 14,762 young (18–23 years) and 14,072 middle-aged (45–50 years) women who were randomly selected from a Medicare database. The participants completed a baseline short-form survey (SF-36), and 12,328 middle-aged women completed a follow-up questionnaire two years later. Young and middle-aged women who reported ever having had ‘low iron’ demonstrated a significantly lower mean physical component summary (PCS), mental component summary (MCS), and vitality (VT) scores (*p* < 0.001), as well as a greater prevalence of ‘constant tiredness’ at baseline compared with women without a history of ID. Additionally, the team reported that after adjustment for the number of children and sociodemographic/clinical variables, mean PCS, MCS, and VT scores in mid-age women at follow-up were lower for those who reported ID in the last two years than for women who reported past ID (prior to two years ago and not in past two years) or no history of ID [16]. 

During reproductive years, chronic blood loss due to the symptom of heavy menstrual bleeding experienced by women has been reported as a major cause of ID [17], rendering women more vulnerable to depleted iron stores and IDA. A typical menstrual cycle results in a blood loss of 25–50 mL and iron loss of 1–3 mg per day [18], and heavy menstrual bleeding is defined as a blood loss of 80 mL per month, with even greater loss of iron [19]. However, heavy menstrual bleeding has remained a largely overlooked contributor to IDA due to the normalization and stigma associated with menstruation in many societies. There is a lack of a structured approach to diagnosing and treating heavy menstrual bleeding, and this further contributes to the frequency of hysterectomies, reflecting the sub-optimal education of gynecologists and disproportionate emphasis on procedural interventions over medical alternatives [13]. 

Low iron stores and anemia do not only represent an individual problem but also an intergenerational one [20,21]. When a woman enters pregnancy in an iron-deficient state, there is an increased likelihood of worsening iron stores. This fosters a cyclical pattern of ID from mother to fetus to child through adolescence and subsequently into the offspring’s pregnancy. While other micronutrient deficiencies, such as vitamin A, B12, and folate, may compound the causal relationship of the untoward health outcomes [22], the diagnosis and treatment of ID/IDA remain a high priority. IDA is associated with a significant increase in mortality and morbidity among reproductive-age non-pregnant women, pregnant women, as well as in their offspring [23]. The compounding effect of anemia is often overlooked in the reporting of many maternal deaths, which are most often attributed to postpartum hemorrhage, hypertensive disorders of pregnancy, infections, and other acute causes [23]. The magnitude of this effect is highlighted by the 1990 estimates that reported that the total mortality burden of over 135,000 deaths annually was directly associated with IDA [7,23]. Several modifiable factors, such as improved diets and access to cleaner environments for infants, children, and women of a reproductive age, can help modify this intergenerational cycle. Low iron stores are also reported to be associated with an increase in postpartum depression, fatigue, and reduced cognition, all impacting a woman’s QOL [24]. 

### 2.3. The Impact of Iron Deficiency, Iron Deficiency Anemia, and Anemia during Pregnancy

There is an increased demand for iron in women throughout pregnancy, with an increase in iron requirements > 6 mg/day in the third trimester [25]. Despite an increase in the absorption of iron during pregnancy, most women are iron-deficient at delivery, especially if they entered the pregnancy in an already iron-deficient state [26]. Consequently, ID in women also affects the accumulation of fetal iron stores at the latter half of the second and during the third trimester of pregnancy, when fetal brain development is accelerated [27]. ID and IDA in pregnancy not only impact maternal outcomes, but fetal underloading has been associated with adverse health and developmental consequences in affected children [28].

A linear relationship exists between the degree of anemia that is diagnosed in the first trimester and subsequent adverse outcomes, consistent across low-, middle-, and high-income countries [29,30,31]. While the rates of maternal anemia tend to be higher in the third trimester, there is an inconsistent correlation with clinical outcomes in the mother and the baby. Anemia that is diagnosed in the first trimester is associated with significantly poorer maternal, fetal, and neonatal outcomes [29,30,31]. There is evidence that a U-shaped curve of reported outcomes may exist when additional iron is provided to iron-replete women to prevent adverse clinical outcomes [32]. 

In a state of ID, iron is prioritized for synthesizing heme at the expense of other essential functions, including fetal organogenesis [11]. Several studies reporting on adverse fetal and neonatal outcomes have noted an increase in stillbirths, low birth weights, and small-for-gestational-age (SGA) babies [33,34]. Other potential adverse effects of maternal ID with or without anemia in children include increased rates of stunting; impairment in both short-term and long-term brain development (brain growth, myelination, neurotransmitters, and brain programming), including significant (likely non-reversible) motor and neurocognitive changes; anxiety and depression; and an increased risk of being on the autism spectrum [35,36,37,38,39].

ID/IDA and anemia have a significant impact on postoperative outcomes, patient recovery, and overall healthcare costs. Richards et al., in 2015, studied 12,836 women undergoing gynecologic surgery within the American College of Surgeons National Surgical Quality Improvement Program database to evaluate the relationship between their preoperative anemia status and the effect of a blood transfusion on postoperative morbidity and mortality. The study reported that approximately one in four (23.9%; CI: 95%, 23.3–24.7) women were anemic while entering surgery and demonstrated an association with an increased risk of 30-day mortality (OR: 2.40; CI: 95%, 1.06–5.44) and composite morbidity (OR: 1.80; CI: 95%, 1.45–2.24) [40]. 

In 2022, a study by Frise et al. reported on the clinical outcomes of ID in 250 patients undergoing non-emergent isolated aortic valve replacement in a tertiary hospital in the United Kingdom. They reported a 22% prevalence of ID and “clinically significant prolongation of total hospital stay (mean increase 2.2 days; 95% CI: 0.5–3.9; *p* = 0.011) and stay within the cardiac intensive care unit (mean increase 1.3 days; 95% CI: 0.1–2.5; *p* = 0.039)”, employing soluble transferrin receptor (sTfR). However, defining ID as plasma ferritin < 100 μg/L levels did not serve as a predictive factor for the length of stay. This study underscores the potential utility of sTfR as a pertinent marker for detecting preoperative non-anemic iron deficiency in patients undergoing cardiac surgery. Moreover, it also suggests the necessity for additional investigations and interventional studies focusing on non-anemic iron deficiency [41]. This considerably increased risk of postoperative adverse outcomes with anemia and ID extends to obstetric surgery. Cesarean delivery rates are increasing worldwide, with increments from 5% to >30% over the past 50 years [42]. Considering that most Caesarean deliveries are elective or emergent, additional opportunities to pre-emptively manage the potential increases in peri- and postoperative complications due to ID, IDA, and anemia should be considered [43,44].

### 2.4. The Diagnosis of Iron Deficiency and Iron Deficiency Anemia among Women

Serum ferritin, an acute-phase reactant, is the best indicator of ID in most individuals. ID is effectively diagnosed by evaluating the serum ferritin level and transferrin saturation (TSAT). Serum ferritin < 30 μg/L and/or TSAT < 20% provides a 98% specificity and 92% sensitivity for absent marrow hemosiderin [33]. IDA can be diagnosed by evaluating the serum ferritin levels and complete blood counts [45]. The current cut-off for anemia is defined as 12 g/dL by the World Health Organization (WHO) [46]. Recent studies have proposed that serum ferritin < 50 μg/L may serve as an indicator of early ID; and this number has been based on the observed correlation of sTfR levels along with the hepcidin ratio [47,48,49]. As evidenced by Frise et al., there is emerging evidence that indicates that existing ferritin thresholds are inappropriately low and may result in underdiagnosis [41]. 

Furthermore, high serum ferritin levels are also seen in functional ID (over 100 μg/L) and may be associated with chronic inflammatory processes that mask ID [49]. Reticulocyte hemoglobin content (Ret-He, sTfR) has recently surfaced as a marker for functional ID. It is a complementary parameter that is especially helpful in cases where ferritin may not be predictive of ID because of an inflammatory state but requires the use of auto-analyzers that may not be available at the point of care [50,51]. 

### 2.5. The Replenishment of Iron in Iron Deficiency and Iron Deficiency Anemia among Women

An increase in dietary intake of iron, even with the concomitant use of multivitamin supplementations, may prove insufficient to address ID/IDA. Various modalities such as the use of new oral iron formulations, parenteral iron supplementation, and blood transfusions are now employed on a global scale to prevent and/or treat ID depending on the severity, underlying cause, and individual patient factors.

### 2.6. Oral Iron as Treatment for Iron Deficiency and Iron Deficiency Anemia in Pregnancy

Globally, oral iron has been the first line of treatment and standard of care to address the prevention and treatment of anemia in pregnancy. In adherent patients, an increase in hemoglobin concentrations and slight decreases in low birthweight rates have been reported. However, 50–70% of pregnant women are non-adherent to a daily dosing due to the untoward side effects of oral iron [52,53]. Even when oral iron is taken as directed in pregnancy, it often does not meet the growing iron demands of anemic mothers and, consequently, their fetuses and offspring. This was demonstrated in a large prospective trial in China, a country with a high rate of mild anemia, showing that even with an increase in the mothers’ hemoglobin concentrations and iron parameters in 45% of cases, there was an insufficient transfer of iron to the fetus and neonate [54].

Published evidence fails to show the benefit of increasing the standard oral iron dosing in anemic pregnant women. From 30 to 60 mg of elemental iron has been reported to provide the same effect on subsequent iron indices as double the dose. These effects are primarily driven by hepcidin, which increases with the dose of oral iron [55]. Alternate-day dosing has been considered to enhance the response of iron supplementation and reduce the side effects of oral iron, including nausea, vomiting, constipation, abdominal discomfort, and black stools [55]. After administering an iron tablet, hepcidin levels are increased, and iron absorption is reduced. The hepcidin level peaks at the eight-hour mark, remains elevated for up to 24 h, and returns to normal at about 48 h, supporting an alternate-day paradigm [56].

A recently published study, from 2023, by von Siebenthal et al. compared daily oral iron provision versus an alternate-day supplementation protocol in 150 young Swiss women with serum ferritin levels of ≤30 μg g/L. The women were divided into two intervention groups in this “double-masked, randomized, placebo-controlled trial”. One group received 100 mg of iron daily for the first 90 days, followed by a daily placebo for an additional 90 days (consecutive-day group). The other group received the same daily iron dose but with a placebo on alternate days for a total of 180 days (alternate-day group). The study reported that when equal total iron doses were administered, the alternate-day dosing group “did not result in higher serum ferritin” compared to the consecutive-day dosing. However, alternate-day dosing did demonstrate a reduction in ID at six months of follow-up, with a lesser likelihood of gastrointestinal side effects [57].

In 2023, in another study published by von Siebenthal et al., the authors evaluated the effect of dietary factors and time of day on the absorption of iron from oral supplements in 34 iron-depleted women. The study participants were administered 100 mg iron doses labeled with different isotopes under six different conditions: “(1) taken with water in the morning; (2) 80 mg ascorbic acid (AA); (3) 500 mg AA; (4) coffee; (5) breakfast including coffee and orange juice (containing  90 mg AA); and (6) water in the afternoon”. Subsequently, the fractional iron absorption was calculated. The study reported that consuming the iron supplement in the morning with orange juice alone resulted in an approximately four-fold increase in iron absorption compared to taking the iron supplementation with coffee or during breakfast. They concluded that the maximum efficacy of oral iron supplementation was reported when ferrous iron supplements were taken in the morning, away from meals or coffee, and preferably with an AA-rich food or beverage [58]. 

### 2.7. Intravenous Iron as a Treatment for Iron Deficiency and Iron Deficiency Anemia in Pregnancy

Although former intravenous (IV) iron supplementation (high-molecular weight dextran) has come with cautionary warnings, and is generally unavailable, newer formulations are now routinely administered in women who do not respond optimally to oral iron supplementation or require rapid normalization of iron stores [59]. IV iron supplementation circumvents the gastrointestinal side effects of oral iron and is responsible for an increase in serum-free iron levels [60]. A number of articles have been published on IV iron supplementation, and it has been proven to be safe in pregnancy beyond 13 weeks of gestation [61,62,63]. Compared to oral iron, IV iron has consistently shown a more rapid and robust response in raising and maintaining hemoglobin concentrations and ferritin during and after delivery [63,64,65].

Newer IV iron formulations such as low-molecular-weight iron dextran, ferumoxytol, ferric carboxymaltose (FCM), and ferric derisomaltose are designed to meet the requirement for an entire course of iron supplementation with a single infusion and with similar safety profiles [66,67]. While safe, other formulations such as ferric gluconate and iron sucrose may require 4–7 infusions, often leading to poor adherence, inappropriate dosing, and a reported increased occurrence of adverse events [68]. Traditionally, both iron sucrose and FCM have served as widely used iron replacement IV formulations. A 2017 trial conducted in India by Sharma et al. demonstrated the superiority of single-dose FCM over iron sucrose [69]. However, these formulations have been associated with adverse outcomes such as rhabdomyolysis with multiple infusions of iron sucrose and high rates of hypophosphatasemia with FCM [70,71]. The clinical relevance of hypophosphatemia with a single infusion of 1 g of iron is presently being studied.

A 2013 study by Filippatos et al. evaluated the impact of FCM independently of anemia in 459 patients with chronic heart failure and ID by randomizing them to FCM or placebo in a 2:1 ratio in the ‘Ferinject^®^ Assessment in patients with IRon deficiency and chronic Heart Failure’ (FAIR-HF trial). The authors reported that the IV iron therapy with FCM in patients with HF and ID was efficacious and safe in improving symptoms and QOL, regardless of anemia status [72].

In 2020, Richards et al. conducted a randomized, double-blind, controlled trial to investigate the impact of IV iron administration on blood transfusions and patient outcomes on anemic individuals undergoing major elective open abdominal surgery. The participants in the intervention arm received a single 1 g dose of ferric carboxymaltose (FCM) given over 15 min in 100 mL normal saline vs. placebo amongst those in the control arm, 10–42 days before surgery. The study found no significant difference between the groups for the composite outcome of blood transfusion or death and number of blood transfusions up to 30 days postoperatively [73].

Talboom et al., in 2023, compared the efficacy of preoperative IV and oral iron supplementation in patients with colorectal cancer and IDA. In a multicenter, open-label, randomized controlled trail, 96 individuals were randomized to the IV iron arm, while 106 were allotted to the oral iron arm. The IV iron group received 1–2 g of FCM for a median of 14 days (interquartile range (IQR) 11–22) before surgery, and the oral iron group received three tablets of 200 mg of oral ferrous fumarate daily for a median of 19 days [IQR 13–27] prior to surgery. The study reported a similar normalization of hemoglobin at the day of admission in both the study groups (RR = 1.08 [95% CI 0.55–2.10]; *p* = 0.83); however, the proportion of patients with normalized hemoglobin significantly increased for the IV iron group later, at 30 days (RR 2.92 [95% CI 1.87–4.58]; *p* < 0.0001). The study did not report any difference in the safety outcomes [74].

In 2023, Pasricha et al. evaluated the effectiveness and safety of a single dose of FCM in the management of anemia in pregnant women of Malawi. The study enrolled anemic women who were in the second trimester of pregnancy (13–26 weeks of gestation) and who were negative for malaria. The participants were randomized to receive either FCM (a single dose of up to 1 g) or the standard of care (60 mg of elemental iron twice daily for 90 days), along with intermittent preventative malaria treatment. The study reported that FCM did not lead to a reduction in anemia prevalence at 36 weeks of gestation compared to the oral iron group (prevalence ratio (PR) 0.92, 95% CI 0.81 to 1.06; *p* = 0.27) or an increase in neonatal birthweight (mean difference −3.1 g [−75.0 to 68.9], *p* = 0.93). FCM was associated with lower anemia levels at all timepoints, significantly at 4 weeks post-treatment (PR 0.91 [0.85 to 0.97]). There were no infusion-related serious adverse effects reported with FCM [75].

In a randomized controlled trial published in 2023, Awomolo et al. investigated whether two infusions of IV ferumoxytol are superior to oral iron supplementation for IDA in pregnancy. The study involved 124 pregnant participants with IDA who were randomly allocated to receive either two infusions of 510 mg of intravenous ferumoxytol spaced approximately seven days apart or taking 325 mg of oral ferrous sulfate twice daily from enrollment until the conclusion of pregnancy. The study demonstrated a statistically significant increase in maternal hemoglobin, hematocrit, iron, and ferritin levels when compared to oral ferrous sulfate supplementation [76]. Further, in another study, Awomolo et al. also investigated the effects of treatment for maternal anemia in neonates. They measured the cord’s blood hematological parameters and reported that offspring born to participants who received IV ferumoxytol exhibited elevated ferritin concentrations, even when matched for gestational age and birth weight. Moreover, participants with elevated hemoglobin and ferritin indices gave birth to infants with increased ferritin concentrations in their cord blood [77].

### 2.8. Postpartum Interventions for Iron Deficiency and Iron Deficiency Anemia

Moya et al. reviewed the existing literature to evaluate the effects of postpartum anemia on maternal health–QOL. The authors reported that ID and/or anemia were a “significant risk factor for postpartum depression and fatigue”, and iron supplementation significantly improved depression and fatigue scores [78]. Even with the use of oral iron supplementation, IDA is common in women during the postpartum period. Despite the paucity of studies, there is emerging evidence assessing treatment options with intravenous iron supplementation to address the high rates of postpartum anemia [61,64,79]. While the systematic review by Markova et al. in 2015 did not report a clear difference on the efficacy of IV iron on postpartum IDA, the authors noted the temporary fatigue score improvement in one study and recommended that the benefits were weighed against the known risks. In contrast, Sultan et al. in 2019 concluded that hemoglobin concentrations at six-weeks postpartum were higher by approximately 1 g/dL in the IV iron group, with reassuring safety profiles [64,80].

Saad et al. recently evaluated the feasibility of administering IV iron following delivery and examined its short-term effect on six weeks postpartum hemoglobin levels in comparison with oral iron. Twenty women with hemoglobin levels that were lower than 9 g/dL were enrolled in each group of this double-blinded randomized controlled trial. They were administered a test dose of IV low-molecular-weight iron dextran infusion. This was followed by either 1000 mg low-molecular-weight iron dextran in 500 mL 0.9% sodium chloride if they had no reaction to the test dose or an IV placebo test dose and infusion. Those who were randomized to IV iron received a six-week supply of placebo tablets, and in contrast, those who were randomized to the placebo infusion received a six-week supply of oral iron (ferrous sulfate 325 mg, 65 mg elemental iron). The study recorded higher median hemoglobin levels at six weeks and beyond in the IV iron group (15 participants) (12.3 [10.6–13.8]) compared to the oral iron group (12 participants) (11.7 [9.9–12.6] g/dL *p* = 0.03). There was no significant difference in the frequency of side effects reported [81]. Larger, on-going studies on the postpartum period, such as the on-going PRIORITY trial, will elicit meaningful outcome data on iron indices and women’s functional status.

Other postpartum interventions have provided an opportunity to intervene early in treating ID and anemia in newborns. Delayed cord clamping appears to be of benefit in improving the iron status and reducing anemia in preterm as well as in term infants [82,83]. In preterm infants, delayed cord clamping improved hemoglobin and serum ferritin at 6 to 10 weeks of life [84]. In term infants, delayed cord clamping significantly improved hemoglobin, ferritin, and TSAT at a variety of time points between 4 and 12 months [85]. However, the duration of wait time for cord clamping remains controversial. The WHO suggests one minute for preterm births and one to three minutes for term deliveries (based primarily on expert opinion), with no adverse impacts on the newborns [84].

### 2.9. Recommendations for Future Research: New Iron Trials Can Potentially Change the Standard of Care

The soon-to-be-completed RAPIDIRON trial is designed to assess the hematologic and clinical differences between pregnant women who are infused with one of two different single-dose IV iron formulations (FCM and ferric derisomaltose, formerly known as iron isomaltoside) and those given oral iron as the standard of care [86,87]. The study is currently being conducted in four geographic regions in India, with a planned recruitment of 4320 women presenting with moderate anemia that has been diagnosed early in pregnancy. Additionally, an economic analysis is being conducted to determine the differential costs based on the mode of delivery, costs linked to adverse maternal and neonatal outcomes, costs associated with an excess length of hospital stay, and differential costs in providing study drugs and the standard of care. The study’s primary outcomes will report on the differences in pregnant women’s return to a non-anemic state at 30–32 weeks of gestation or just prior to delivery, with a second primary clinical outcome reporting on rate differences in low birth weight.

RAPIDIRON-KIDS, another on-going study, is a three-year follow-up trial of a subset of children whose mothers were randomized in RAPIDIRON, with rates of maternal anemia recorded at the time of delivery from cord blood and at four months postdelivery. Additionally, the differences in Bayley Scales of Infant and Toddler Development (BSID) scores will be measured in the children of women randomized to RAPIDIRON at two years. In an ancillary Neuroimaging Study conducted on a convenience subset of the study population, morphological changes during the development of fetal and children’s brains using magnetic resonance imaging (MRI) will be studied to explore its correlations to RAPIDIRON-KIDS outcomes.

Additional studies examining individual IV iron formulations with a focus on their indications and adverse effects in preventing and treating ID/IDA in reproductive-age women in low- and middle-income countries are needed.

## 3. Conclusions

Several decades have elapsed since oral iron supplementation has been utilized as the standard of care in treating and preventing ID and IDA. Nevertheless, the reported results have been sub-optimal. The extensive benefits of iron replenishment are underutilized due to the recorded side effects of oral supplementation that lead to poor compliance. Though a slight improvement in the rates of anemia has been noted over the years, many babies are still being born with ID and suffer adverse sequelae due to inadequate iron replenishment in the mothers. Perhaps it is time to consider a broad scale-up of single-dose IV iron supplementation as a therapeutic intervention and establish a new standard of care to address ID/IDA. However, a change in practice will only occur when such an intervention is proven to be feasible, safe, efficacious, and cost-effective.

## Data Availability

No new data were created.
